# Impact of mineral fertilization and *Trichoderma* application on soil microbiota of young olive trees inoculated with *Verticillium dahliae*

**DOI:** 10.3389/fmicb.2025.1708981

**Published:** 2025-11-25

**Authors:** Guillem Segarra, Marc Sancho-Adamson, M. Isabel Trillas, Joan Romanyà

**Affiliations:** 1 Serra Húnter Fellow, Plant Physiology Section, Department of Evolutionary Biology, Ecology and Environmental Sciences, Faculty of Biology, University of Barcelona, Barcelona, Spain; 2 Plant Physiology Section, Department of Evolutionary Biology, Ecology and Environmental Sciences, Faculty of Biology, University of Barcelona, Barcelona, Spain; 3 Agrobiology and Soil Management, Department of Biology, Health and Environment, Faculty of Pharmacy and Food Sciences, University of Barcelona, Barcelona, Spain; 4 Institute of Nutrition and Food Safety (INSA-UB), University of Barcelona, Barcelona, Spain; 5 CIBER Physiopathology of Obesity and Nutrition (CIBEROBN), Institute of Health Carlos III, Madrid, Spain

**Keywords:** *Trichoderma*, Verticillium, fertilization, olive tree, soil microbiota

## Abstract

**Introduction:**

*Verticillium dahliae*, the pathogen producing Verticillium wilt in olive orchards is a soilborne pathogenic fungus that has a long persistence in soil due to the formation of melanized microsclerotia and represents a devastating threat to the production in Mediterranean countries. Management of Verticillium wilt of olive is not easily achieved by means of a single treatment and thus integrated approaches are needed. *Trichoderma asperellum* strain T34 is a biological control agent that was isolated from a suppressive compost and has been shown to reduce the severity of various soil-borne diseases in many crops.

**Material and methods:**

Two-year-old olive trees were planted in pots containing soil. Plants were subjected to 3 factors (fertilization, inoculation with the pathogen *Verticillium dahliae* and *Trichoderma* application) each one with two levels (yes or no), resulting in 8 groups (treatments) of plants. Soils were sampled 20 months after transplanting to perform 16S and ITS sequencing as well as to quantify the concentration of *V. dahliae* microsclerotia.

**Results:**

The treatment of the pots with the biological control agent *T. asperellum* strain T34 effectively reduced the amount of *V. dahliae* microsclerotia, suggesting a promising alternative to chemical fumigation. Moreover, it did not affect the diversity of bacteria and fungi in the rhizospheric soil of olive trees. On the other hand, mineral fertilization doubled the amount of microsclerotia in soil and drastically increased the relative abundance of *V. dahliae* reads. Furthermore, fertilization had a significant effect on microbial communities, mostly on bacterial populations. Interestingly, fertilization did not have an effect on the phylum Glomeromycota, and bacterial genera affected by fertilization were not specifically associated to N fixing or non-N fixing bacteria.

**Conclusion:**

Taken together, those results suggest that mineral fertilization has a much more profound impact on the relative abundance of microorganisms than the introduction of biological control agents such as *T. asperellum* strain T34.

## 1 Introduction

*Verticillium dahliae* is the pathogen producing Verticillium wilt in olive orchards and means a devastating threat to the production in Mediterranean countries (López-Escudero and Mercado-Blanco, 2011). It is a soilborne pathogenic fungus that has long persistence in soil even in the absence of a host due to the formation of melanized microsclerotia (Short et al., 2015). Favorable environmental conditions and the presence of root exudates stimulate microsclerotia germination and hyphae penetrate roots and colonize the epidermal cells and cortex (Jiménez-Díaz et al., 2012). Once vascular colonization occurs, and conidia formed are transported upwards via xylem, both fungus and plant reactions lead to partial block of xylem vessels which are responsible for chlorosis and wilt of the leaves (Trapero et al., 2018). Among other causes, Verticillum wilt of olive has increased in the last decades due to the introduction of intensive cultivation systems in olive orchards. It is assumed that, similarly to what has been reported in cotton, excessive N fertilization and high irrigation doses typical from intensive cultivation will increase the severity of *V. dahliae* infections (López-Escudero and Mercado-Blanco, 2011). Management of Verticillium wilt of olive is difficult to achieve by using a single treatment. For instance, an integrated approach has been proposed that combines preplanting and postplanting control measures including avoidance of highly infested soils, using pathogen free plants, reduction of inoculum in soil, use of resistant cultivars and agronomic practices (Jiménez-Díaz et al., 2012). Chemical fumigation with methyl bromide, now banned, to reduce *V. dahliae* microsclerotia in soils was a common practice for decades. Alternative treatments such as the application of organic amendments and biological control are interesting from the point of view of safety and environmental impact. Significant reduction in microsclerotia viability and the severity of the symptoms of *V. dahliae* in olive trees were obtained by using grape marc compost and solid olive-oil waste, combined with other organic amendments (Varo-Suárez et al., 2018). When studying the ability of several olive mill composts to suppress *V. dahliae*, it was shown that enzymatic diversity, b-glucosidase activity, pH, and electrical conductivity may be sufficient to predict if plant growth media amended with a given compost will be suppressive to Verticillium wilt (Avilés and Borrero, 2017). Moreover, the non-pathogenic strain of *Fusarium oxysporum* FO12 was effective in reducing soil inoculum and also reduced the incidence of Verticillium wilt in olive trees (Mulero-Aparicio et al., 2020) suggesting the potential for biological control in the management of this disease.

*Trichoderma asperellum* strain T34 is a biological control agent that was isolated from a suppressive compost and has been shown to reduce severity of diseases produced by the soil-borne pathogen *Fusarium oxysporum* in various crops (Sant et al., 2010; Segarra et al., 2010). Furthermore, it has been shown to induce systemic resistance against foliar diseases such as *Botrytis cinerea* and *Hyaloperonospora parasitica* when applied to the roots (Segarra et al., 2009; Fernández et al., 2014; Martínez-Medina et al., 2017). *T. asperellum* strain T34 is an authorized active substance to be used as plant protection product. Contrary to chemical plant protection products, biological control agents may have more than one mode of action, including parasitism, competition for nutrients, production of metabolites and even the induction of plant defenses (Köhl et al., 2019). For instance, studies with *T. asperellum* strain T34 indicate that competition for Fe and parasitism are present in the antagonism against *F. oxysporum* while induction of resistance is the main mode of action against foliar diseases (Segarra et al., 2010).

Given that microorganisms used in biological control are usually introduced in the environment, for example in the soil, at concentrations higher than the natural concentrations, one of the aspects that has to be proven is that it does not alter significantly the natural microbial populations which play a critical role in driving essential soil functions such as nutrient cycling, organic matter decomposition, soil structure and plant health (Kaminsky et al., 2019; Köhl et al., 2019; Chen et al., 2024).

Intolerable non-target effects could be defined as those that persist beyond the time of crop harvest and that are significantly different from changes produced by the growth of the plant and agricultural practices (Winding et al., 2004). In this sense, agricultural management practices such as fertilization have been reported as an important factor disturbing microbial populations in the soil (Mishra et al., 2022). It is usually accepted that increases in nutrient availability tend to promote copiotrophic microbial taxa which exhibit fast growth and low C use efficiency while reduces the abundances oligotrophic taxa with slow growth and high C use efficiency (Leff et al., 2015). Indeed, fitness of soil saprotrophic fungal taxa is expected to be low in low C agricultural soils (Bonner et al., 2022).

Taken all together we hypothesized that agricultural management practices such as fertilization might have a higher impact on soil microbial populations than the application of a potential biological control agent in potted olive trees grown in soil obtained from a well stablished productive olive orchard. Specifically, our aims were i) to study the potential of *T. asperellum* strain T34 to control Verticillium wilt of olive and ii) to study the impact of the introduction of the biological control agent *T. asperellum* strain T34 on the soil microbial populations in potted olive trees compared to the effect of mineral fertilization.

## 2 Materials and methods

### 2.1 Greenhouse experiment

Two-year-old olive tree clones (*Olea europaea* L.) of the cultivar Picual were planted in 10-L pots containing soil. The soil used was a sandy loam (14.5% clay) described as a Calcaric Cambisol with an organic C content of 0.86%, C/N ratio of 10.7, and pH of 8.58 and was collected from a commercial olive orchard (Romanyà et al., 2019). Before potting, the soil was sieved and mixed with perlite at the ratio of 2 soil:1 perlite (v/v) in order to improve aeration. A greenhouse experiment was set up at the Torribera Campus of the University of Barcelona, where environmental conditions were controlled by opening and closing the roof. Air temperatures ranged from 6 to 30 °C and relative humidity ranged from 11 to 59%. Plants were watered according to demand by drip irrigation in order to maintain field capacity. Plants were subjected to 3 factors (fertilization, inoculation with the pathogen *Verticillium dahliae* and *Trichoderma* application) each one with two levels (yes or no), resulting in 8 groups (treatments) of plants. Each treatment included 10 replicates. All treatments were randomly distributed on greenhouse benches.

Fertilization was applied to pots designated as F+ as an NPK fertilizer (ENTEC Nitrofoska 14–7–17, EuroChem Agro, Barcelona, Spain) that contained 8% ammonia-N and 6% nitrate-N, 7% P_2_O_5_, 17% K_2_O, 22.5% SO_3_, 2%MgO, 0.02% B, 0.01% Zn, and 0.8% 3,4-dimethylpyrazole phosphate (DMPP) at the dose of 142.5 kg N ha^–1^, 31.1 kg P ha^–1^ and 150.45 kg K ha^–1^. Pots not treated with fertilizer were designated as F-.

*Trichoderma* application was performed by applying the biocontrol agent *Trichoderma asperellum* strain T34 (commercially available as T34 Biocontrol^®^). It was inoculated with a conidial suspension to a final concentration of 1 × 10^4^ colony forming units (CFU) per ml of soil. Along the experiment, plants received 4 applications of T34: 2, 6 (spring), 12 (fall) and 18 (spring) months after transplantation, Plants that received *Trichoderma* application were designated T+, while plants not treated were designated as T-.

A defoliating pathotype of *Verticillium dahliae* was kindly provided by Dr. Manuel Avilés (Avilés and Borrero, 2017). Pathogen inoculation was performed by applying a conidial suspension to a final concentration of 10^6^ CFU/mL soil (Romanyà et al., 2019). The conidial suspension was produced in a 40 L fermenter with Czapek-Dox Broth during 5 days with the following conditions: aeration rate of 6 l min−1 (pO_2_ adjusted at 100 ± 5%), agitation of 1,000 rpm, 25 °C and non-buffered pH. Inoculated plants were designated as V+ and received a total of 4 applications of the pathogen, which were performed 1 week after each application of the biological control agent. Non inoculated plants were designated as V-.

### 2.2 Nutrient analysis

Three weeks after the first *V. dahliae* inoculation 5 plants per treatment were randomly selected for nutrient analysis. Two mature leaves (present at the moment of transplanting) and two young leaves (formed after transplanting) were sampled from each selected plant. Three months after the first inoculation leaves opposite to the leaf sampled as a young leaf were sampled and considered to be mature leaves; new grown young leaves at the tip of each selected shoot were also sampled.

Leaves were rinsed with deionized water, oven-dried at 70 °C for 48 h and weighed. Samples were finely ground in an agate mortar. N content was determined by elemental analyzer (Thermo EA 108 CHNS-O, Carlo Erba Instruments). A 10–40 mg of ground plant tissue was pre-treated with nitric acid (HNO_3_ 69.5%) and left overnight. On the following day, all tubes were heated at 80 °C for an hour, let cool, and then 0.5 ml of perchloric acid (HClO_4_ 70%) was added before heating the samples to 180 °C for 3 h. Samples were then filtered and made up to 10 ml volume with deionised water. Induced coupled plasma optical emission spectrometry (simultaneous ICP-OES, Perkin Elmer Optima 8300) was used to determine element content (P, K, S, Ca, Mg and Fe) in the extracts.

### 2.3 Analysis of the number of sclerotia present in the soil

Soils were sampled 20 months after transplanting and processed as previously described in order to quantify the concentration of *V. dahliae* microsclerotia (Avilés and Borrero, 2017). 3 pots per treatment were sampled. Briefly, 25 gram of the soil sample was suspended in 250 ml of distilled water and agitated during 1 h at 270 rpm. The suspension was filtered through nested 150 and 36 μm sieves with tap water and the material retained in the 36 μm sieve was recovered, made up to 100 ml with distilled water and plated on modified soil extract agar medium (Harris et al., 1993). Two weeks after, the residues were eliminated from the surface of the plates, and they were dried and incubated for 2–3 additional weeks after which *V. dahliae* colonies were counted.

### 2.4 DNA extraction and sequencing

Soils were sampled 20 months after transplanting using a 1.4-cm diameter auger. Eight subsamples from each pot were combined into one sample and roots were separated manually. Samples were stored at 4 °C before DNA extraction. 3 pots per treatment were sampled. One gram of each soil sample was used to extract DNA using the E.Z.N.A.™ Soil DNA isolation kit according to the manufacturer instructions. The quality and quantity of DNA was checked spectrophotometrically using a NanoPhotometer P-Class (Implen GmbH, Germany). The amplification was performed at MR DNA (Shallowater, TX, United States) using the primers illCUs515F GTGYCAGCMGCCGCGGTAA and new806RB GGACTACNVGGGTWTCTAAT for bacteria (16S rRNA gene V4 variable region) and ITS1F-Bt1 CTTGGTCATTTAGAGGAAGTAA and ITS2R GCTGCGTTCTTCATCGATGC for fungi (ITS1 gene) (Walters et al., 2016). A PCR consisting of 94 °C for 3 min, followed by 30 cycles of 94 °C for 30 s, 53 °C for 40 s, 72 °C for 1 min, and a final elongation step at 72 °C for 5 min was performed with the HotStarTaq Plus Master Mix Kit (Qiagen, United States). The success of amplification and the relative intensity of the bands was checked in 2% agarose gel. Samples were pooled together in equal proportions based on their molecular weight and DNA concentrations, they were purified using calibrated AMPure XP beads and the Illumina TruSeq Nano DNA library was prepared. The Illumina MiSeq sequencing platform at MR DNA was used according to the manufacturer’s instructions. The standardized analysis pipeline consisted in the joining of the sequences, barcode depletion, removal of short sequences (<150 bp) and removal of sequences with ambiguous base calls (Chiodini et al., 2015). After the sequences were denoised the operational taxonomic units (OTUs) were generated and UCHIME was used to remove chimeras. UCLUST in standard default was used to define OTUs, after the removal of singleton sequences, by clustering at 97% similarity. BLASTn was used against a curated database derived from RDPII^1^ and NCBI^2^ databases to taxonomically classified final OTUs. The sequence data generated in this study were deposited in the NCBI Sequence Read Archive under BioProject ID PRJNA628525. Fungal genera were classified by trophic modes according to the FUNGuild database (Nguyen et al., 2016). Bacterial genera found to be significantly affected by the factors were classified as N fixing or not according to Nelson et al. (2016).

### 2.5 Statistical analysis

Microbiome analyst was used to run principal coordinates analyses (PCoA) using a Bray–Curtis dissimilarity matrix on the OTU data as well as PERMANOVA (Chong et al., 2020). The effect of the factors fertilization, *Trichoderma* application and *Verticillium* inoculation on the relative abundance of bacteria and fungi were studied at genus and phylum levels on squared root- transformed data by means of a 3-way ANOVA performed on IBM SPSS Statistics for Windows Version 21.0 (IBM Corp.) and the *p*-values were corrected to consider the false discovery rate (Benjamini and Hochberg, 1995). Krona was used to represent taxonomical distribution of bacteria and archaea genera affected by fertilization (Ondov et al., 2011).

## 3 Results

During the course of the experiment, despite receiving 4 applications of the pathogen, inoculated plants remained asymptomatic, presenting no evident signs of wilt or defoliation.

**FIGURE 1:**
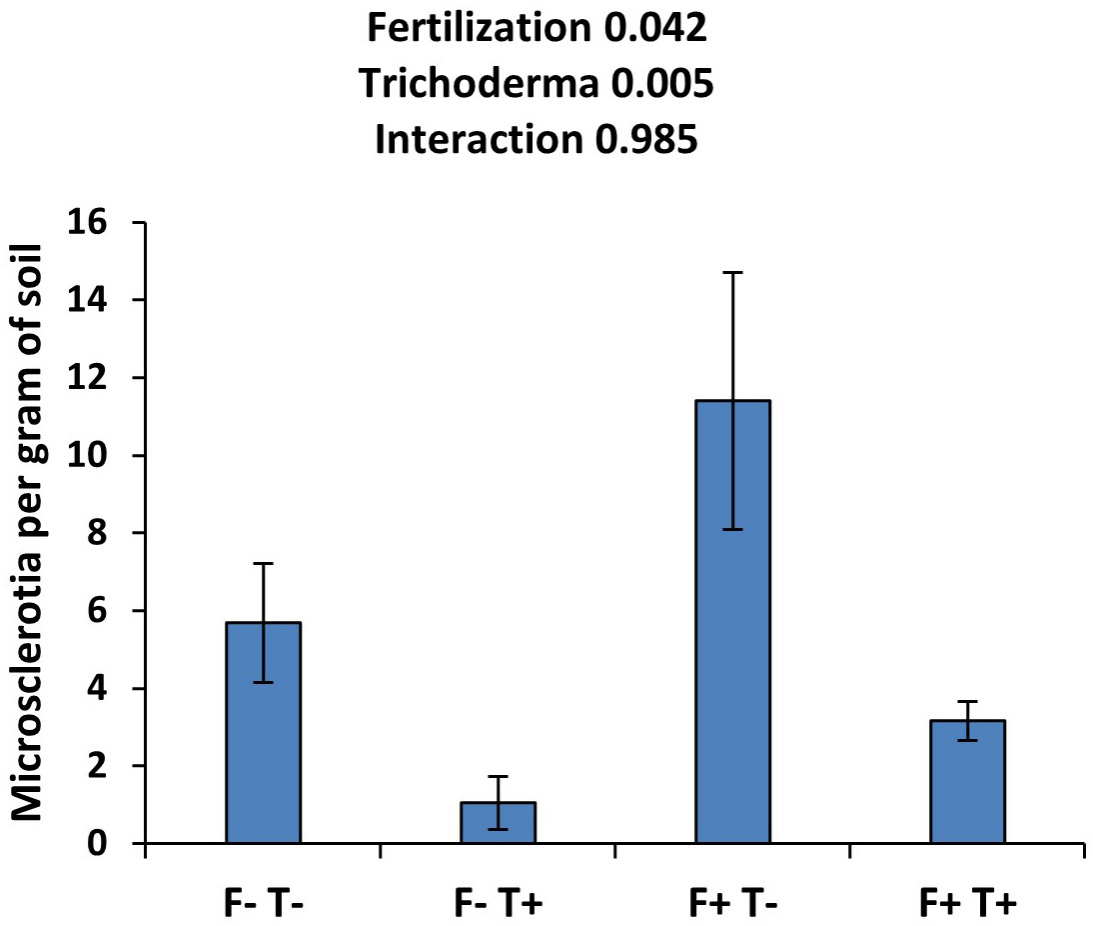
Effect of fertilization and *T. asperellum* strain T34 application on the concentration of microsclerotia in the soil. F+ and F-, fertilized and not-fertilized pots, respectively. T+ and T-, treated or not with T34. Means ± standard error of the means are shown. The number next to the name of the factor indicates the *p*-value.

The effect of fertilization and *Trichoderma* application on the number of sclerotia per gram of soil is shown in Figure 1. The fertilization of the plants resulted in a significant increase in the concentration of microsclerotia in the soil compared to not fertilized plants. On the other hand, the application of the biological control agent *T. asperellum* strain T34 resulted in a significant decrease in microsclerotia concentration. The interaction of both factors was not significant suggesting that the beneficial effect of T34 is independent of the fertilization. The concentration of microsclerotia doubled in the presence of fertilization and the absence of T34. T34 application resulted in an 82% reduction in the concentration of microsclerotia in not fertilized plants and a 72% reduction in fertilized plants.

**FIGURE 2:**
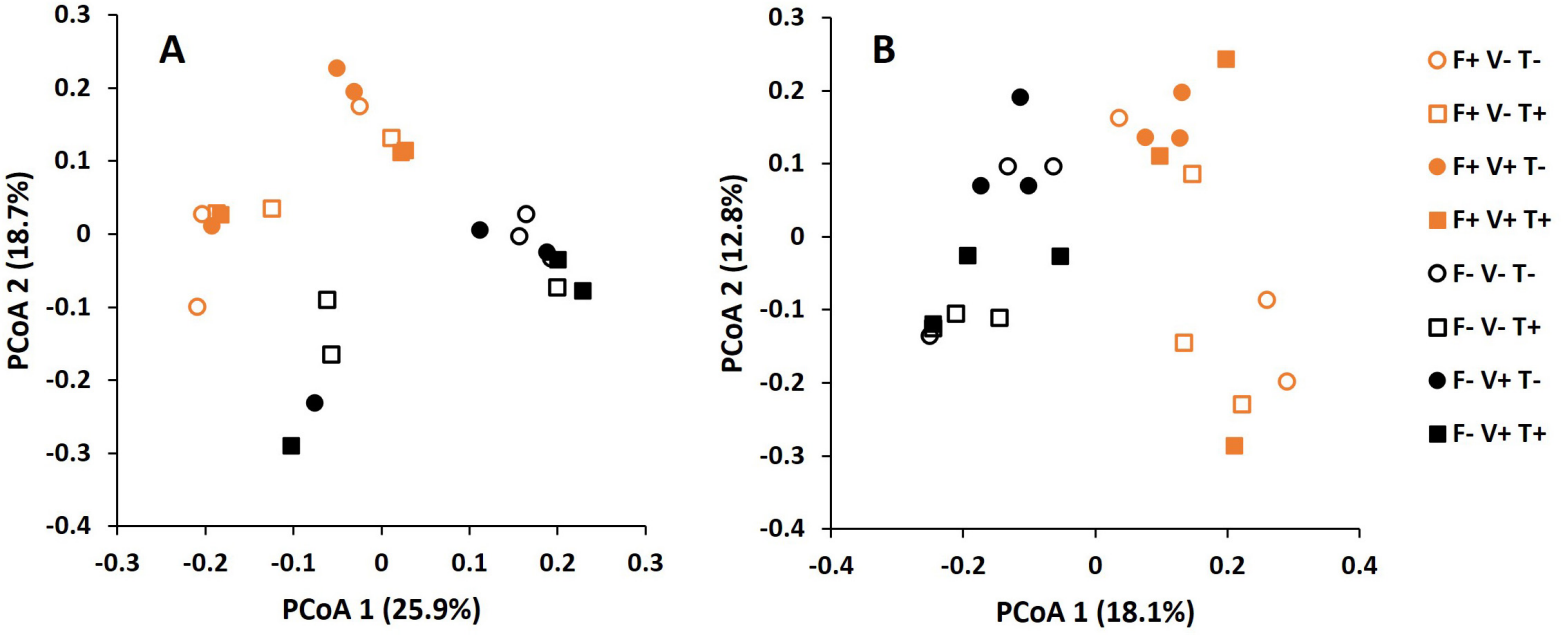
Principal coordinate analysis (PCoA) of bacterial **(A)** and fungal **(B)** operational taxonomic units (OTUs) in the soil subjected to the factors fertilization, *V. dahliae* inoculation and T34 application.

The Permanova analysis revealed that the effect of fertilization on soil bacterial microbiome was significant (*F*-value: 5.473; *p* = 0.001) while the effect of T34 application and *V. dahliae* inoculation was not significant (Figure 2A). The component 1 and 2 of the PCoA explained a 25.9 and 18.7% of variation, respectively. Similarly, the Permanova analysis of fungal OTUs showed that the effect of fertilization was significant (*F*-value: 4.164; *p* = 0.001) while the factors T34 application and *V. dahliae* inoculation were not significant (Figure 2B). For fungi, the component 1 and 2 of the PCoA explained an 18.1 and 12.8% of variation, respectively.

Alpha diversity indexes such as Richness and Shannon did not show significant differences due to fertilization, pathogen inoculation or *Trichoderma* application (Table 1).

**TABLE 1:** Alpha diversity (Richness and Shannon index) at OTU level of soil microbiota subjected to the factors fertilization, *V. dahliae* inoculation and *Trichoderma* application.

Treatment	Bacteria and archaea		Fungi	
	Richness	Shannon	Richness	Shannon
F-V-T-	3442.0 ± 89.2	6.9 ± 0.0	739.6 ± 37.5	4.5 ± 0.1
F-V-T+	3424.0 ± 101.1	6.6 ± 0.0	741.0 ± 60.1	4.6 ± 0.0
F-V+T-	3271.0 ± 140.3	6.5 ± 0.2	717.3 ± 15.0	4.2 ± 0.1
F-V+T+	3239.0 ± 43.0	6.4 ± 0.2	725.0 ± 42.2	4.3 ± 0.2
F+V-T-	3193.3 ± 75.2	6.4 ± 0.3	727.6 ± 82.0	4.3 ± 0.2
F+V-T+	3264.6 ± 126.2	6.6 ± 0.1	748.6 ± 31.4	4.5 ± 0.1
F+V+T-	3261.3 ± 45.2	6.6 ± 0.2	777.0 ± 48.4	4.6 ± 0.1
F+V+T+	3204.0 ± 199.3	6.6 ± 0.2	697.3 ± 51.6	4.1 ± 0.3

Values represent the mean ± standard error of the mean. There were no significant differences among factors and interactions in a 3-way ANOVA. Fertilization: yes, F+; no, F-. *V. dahliae* inoculation: yes, V+; no, V-. *Trichoderma* application: yes, T+; no, T-. See ANOVA table in Supplementary material.

The relative abundance of all bacterial and archaeal phyla present in the soil samples is shown in Table 2. The most abundant phyla were, in order, Proteobacteria, Actinobacteria and Firmicutes, which accounted for a relative abundance of 77.75% in non-treated soils. The relative abundance of Actinobacteria, Candidatus Tectomicrobia, Firmicutes increased when soil was fertilized, while the relative abundance of Elusimicrobia, Fibrobacteres, Nitrospinae, Nitrospirae, Proteobacteria, Spirochaetes and Tenericutes decreased. Neither the inoculation with the pathogen nor the treatment with the biological control agent affected the relative abundances of bacterialphyla. In our study the Archaea phylum found in the samples were Euryarchaeota and Thaumarchaeota and their relative abundances were not affected by the treatments.

**TABLE 2:** Relative abundance (%) of all bacterial and archaeal Phyla present in the samples according to the treatments they received.

Phyla	No fertilization				Fertilization			
	No Verticillium		Verticillium		No Verticillium		Verticillium	
	No *Trichoderma*	*Trichoderma*	No *Trichoderma*	*Trichoderma*	No *Trichoderma*	*Trichoderma*	No *Trichoderma*	*Trichoderma*
	F-V-T-	F-V-T+	F-V+T-	F-V+T+	F+V-T-	F+V-T+	F+V+T-	F+V+T+
Acidobacteria	3.39 ± 0.21	2.88 ± 0.14	2.98 ± 0.33	2.6 ± 0.34	2.74 ± 0.23	2.77 ± 0.18	2.66 ± 0.15	2.62 ± 0.22
**Actinobacteria^1^**	**15.78 ± 1.22**	**13.21 ± 1.08**	**11.77 ± 1.84**	**11.12 ± 1.78**	**19.38 ± 3.24**	**17.99 ± 1.27**	**25.9 ± 2.37**	**18.41 ± 1.33**
Armatimonadetes	0.05 ± 0.02	0.04 ± 0.02	0.02 ± 0.01	0.01 ± 0.01	0.00 ± 0.00	0.00 ± 0.00	0.00 ± 0.00	0.02 ± 0.01
Bacteroidetes	3.95 ± 0.76	2.97 ± 0.24	3.32 ± 0.8	3.64 ± 0.53	2.74 ± 0.15	3.68 ± 0.39	2.36 ± 0.21	3.35 ± 0.36
**Candidatus Tectomicrobia ^1^**	**0.21 ± 0.02**	**0.16 ± 0.02**	**0.18 ± 0.02**	**0.16 ± 0.01**	**0.25 ± 0.01**	**0.21 ± 0.02**	**0.22 ± 0.02**	**0.18 ± 0.01**
Chlamydiae	0.05 ± 0.00	0.04 ± 0.00	0.04 ± 0.01	0.03 ± 0.01	0.03 ± 0.01	0.03 ± 0.01	0.04 ± 0	0.03 ± 0.01
Chloroflexi	3.06 ± 0.1	3.17 ± 0.36	2.37 ± 0.43	2.12 ± 0.16	3.39 ± 0.41	3.26 ± 0.37	3.08 ± 0.19	2.51 ± 0.21
Cyanobacteria	0.88 ± 0.18	0.72 ± 0.14	1.08 ± 0.36	1.03 ± 0.09	0.78 ± 0.18	1.08 ± 0.66	0.5 ± 0.10	0.87 ± 0.26
Deinococcus-Thermus	0.2 ± 0.01	0.23 ± 0.02	0.21 ± 0.03	0.26 ± 0.04	0.26 ± 0.02	0.24 ± 0.04	0.28 ± 0.08	0.24 ± 0.03
**Elusimicrobia ^1^**	**0.07 ± 0.02**	**0.04 ± 0.02**	**0.05 ± 0.02**	**0.05 ± 0.01**	**0.01 ± 0.00**	**0.01 ± 0.00**	**0.01 ± 0.00**	**0.01 ± 0.00**
Euryarchaeota	0.04 ± 0.01	0.05 ± 0	0.05 ± 0.01	0.03 ± 0.00	0.02 ± 0.01	0.05 ± 0.01	0.03 ± 0.01	0.08 ± 0.04
**Fibrobacteres^1^**	**0.19 ± 0.04**	**0.25 ± 0.05**	**0.17 ± 0.01**	**0.2 ± 0.03**	**0.07 ± 0.02**	**0.09 ± 0.01**	**0.06 ± 0.01**	**0.15 ± 0.06**
**Firmicutes^1^**	**5.13 ± 0.09**	**6.26 ± 0.64**	**5.39 ± 0.52**	**5.04 ± 0.76**	**8.88 ± 0.83**	**12.1 ± 4.97**	**11.13 ± 1.35**	**7.19 ± 1.28**
Fusobacteria	0.01 ± 0	0.01 ± 0	0.01 ± 0	0.01 ± 0.00	0.01 ± 0.00	0.01 ± 0	0.01 ± 0.00	0.01 ± 0.01
Gemmatimonadetes	4.14 ± 0.09	3.35 ± 0.32	3.35 ± 0.45	3.25 ± 0.65	3.78 ± 0.51	3.46 ± 0.47	3.95 ± 0.41	3.77 ± 0.62
Ignavibacteriae	0.07 ± 0.03	0.04 ± 0.01	0.07 ± 0.03	0.06 ± 0.02	0.03 ± 0.00	0.07 ± 0.01	0.03 ± 0.01	0.04 ± 0.01
**Nitrospinae ^1^**	**0.02 ± 0.01**	**0.01 ± 0.00**	**0 ± 0.00**	**0.01 ± 0.01**	**0.00 ± 0.00**	**0.00 ± 0.00**	**0.00 ± 0.00**	**0.00 ± 0.00**
**Nitrospirae^1^**	**0.49 ± 0.04**	**0.38 ± 0.03**	**0.37 ± 0.04**	**0.43 ± 0.04**	**0.33 ± 0.05**	**0.31 ± 0.07**	**0.27 ± 0.00**	**0.25 ± 0.04**
Planctomycetes	2.9 ± 0.09	2.63 ± 0.02	2.46 ± 0.27	2.31 ± 0.23	2.44 ± 0.03	2.52 ± 0.11	2.98 ± 0.27	2.66 ± 0.23
**Proteobacteria ^1^**	**56.84 ± 1.5**	**61.06 ± 2.2**	**63.86 ± 4.06**	**65.72 ± 2.2**	**52.43 ± 2.92**	**49.64 ± 2.3**	**44.1 ± 2.25**	**55.7 ± 2.03**
**Spirochaetes^1^**	**0.03 ± 0.00**	**0.02 ± 0.01**	**0.02 ± 0.01**	**0.02 ± 0.01**	**0.01 ± 0.00**	**0.02 ± 0.01**	**0.01 ± 0.00**	**0.01 ± 0.01**
**Tenericutes^1^**	**0.09 ± 0.01**	**0.03 ± 0.02**	**0.04 ± 0.01**	**0.04 ± 0.02**	**0.02 ± 0.01**	**0.02 ± 0.00**	**0.03 ± 0.02**	**0.02 ± 0.01**
Thaumarchaeota	1.35 ± 0.15	1.58 ± 0.15	1.2 ± 0.12	0.99 ± 0.17	1.59 ± 0.01	1.35 ± 0.2	1.55 ± 0.07	0.98 ± 0.15
Verrucomicrobia	1.04 ± 0.04	0.84 ± 0.07	0.98 ± 0.08	0.85 ± 0.04	0.82 ± 0.11	1.09 ± 0.13	0.81 ± 0.15	0.9 ± 0.12

^1^The factor Fertilization is significant for this Phylum. Factor significance is indicated by bold text. No other significant factors or interactions were found in a 3-way ANOVA including factors Fertilization, *Verticillium*, *Trichoderma* and all possible interactions. Values represent the mean ± standard error of the mean. See ANOVA table in Supplementary material.

The relative abundance of all fungal phyla present in the soil samples is shown in Table 3. The most abundant phyla were, in order, Ascomycota, Basidiomycota and Glomeromycota that accounted for a relative abundance of 84.06% in non-treated soils. The relative abundance of Mucoromycotina decreased when soil was fertilized. The factor *Verticillium* and the interaction of factors Fertilization × *Trichoderma* were significant for the phylum Blastocladiomycota. Above all, no treatment affected significantly the relative abundance of Blastocladiomycota compared to the untreated control. However, when fertilization was present, the application of *Trichoderma* and/or the inoculation with *Verticillium* significantly decreased the relative abundance of this phylum compared to the fertilized control.

**TABLE 3:** Relative abundance of all fungal phyla found in the samples according to the combination of treatments they received.

Phyla	No Fertilization				Fertilization			
	No *Verticillium*		*Verticillium*		No *Verticillium*		*Verticillium*	
	No *Trichoderma*	*Trichoderma*	No *Trichoderma*	*Trichoderma*	No *Trichoderma*	*Trichoderma*	No *Trichoderma*	*Trichoderma*
	F-V-T-	F-V-T+	F-V+T-	F-V+T+	F+V-T-	F+V-T+	F+V+T-	F+V+T+
Ascomycota	54.45 ± 2.83	41.59 ± 3.19	50.85 ± 1.08	57.12 ± 8.4	50.72 ± 4.16	48.64 ± 5.11	62.52 ± 8.45	57.65 ± 6.07
Basidiomycota	20.28 ± 3.63	40.82 ± 6.09	22.09 ± 4.64	25.57 ± 6.61	25.52 ± 8.7	33.05 ± 5.53	20.31 ± 6.42	23.94 ± 12.32
**Blastocladiomycota ^1^**	**0.44 ± 0.12 abc**	**0.62 ± 0.07 ab**	**0.13 ± 0.03 c**	**0.31 ± 0.04 abc**	**0.94 ± 0.43 a**	**0.17 ± 0.03 bc**	**0.19 ± 0.02 bc**	**0.07 ± 0.01 c**
Chytridiomycota	3.06 ± 0.69	4.05 ± 1.44	2.99 ± 1.76	2.72 ± 1.28	6.26 ± 0.73	4.04 ± 1.53	2.6 ± 0.98	2.06 ± 0.72
Cryptomycota	0.11 ± 0.04	0.02 ± 0.01	0.04 ± 0.02	0.88 ± 0.87	0.02 ± 0.01	0.01 ± 0	0.03 ± 0.01	0.03 ± 0.01
Entomophthoromycota	0.03 ± 0.01	0.06 ± 0.03	0.02 ± 0.01	0.02 ± 0.02	0.04 ± 0.04	0.03 ± 0.02	0.04 ± 0.03	0.06 ± 0.03
Glomeromycota	9.33 ± 2.06	5.01 ± 1.43	11.07 ± 2.52	5.24 ± 2.16	8.59 ± 4.05	7.67 ± 1.24	9.46 ± 1.68	8 ± 5.36
Kickxellomycotina	0.16 ± 0.03	0.1 ± 0.03	0.21 ± 0.02	0.14 ± 0.05	0.16 ± 0.05	0.07 ± 0.02	0.5 ± 0.39	0.11 ± 0.04
Mortierellomycotina	3.6 ± 1.74	4.74 ± 1.66	1.42 ± 0.94	1.06 ± 0.51	6.73 ± 1.39	3.93 ± 1.32	3.55 ± 0.66	7.54 ± 3.51
**Mucoromycotina ^2^**	**8.37 ± 3.32**	**2.83 ± 1.4**	**11.12 ± 2.8**	**6.9 ± 1.8**	**0.91 ± 0.43**	**2.34 ± 0.83**	**0.79 ± 0.49**	**0.51 ± 0.38**
Olpidiaceae	0.18 ± 0.06	0.15 ± 0.12	0.06 ± 0.03	0.04 ± 0.01	0.1 ± 0.09	0.05 ± 0.03	0.02 ± 0.01	0.03 ± 0.01

^1^ The factor *Verticillium* and the interaction of factors Fertilization x *Trichoderma* are significant for this phylum. Different letters indicate significant differences between different treatment combinations. ^2^The factor Fertilization is significant for this phylum. Factor significance is indicated by bold text. No other significant factors or interactions were found in a 3-way ANOVA including factors Fertilization, *Verticillium*, *Trichoderma* and all possible interactions. Values represent the mean ± standard error of the mean. See ANOVA table in Supplementary material.

Fertilization affected either positively or negatively the relative abundance of 162 genera of bacteria and archaea in a significant way (Table 4). By contrast, T34 had no effect on any genera of bacteria or archaea. The phylum with the major number of genera affected was Proteobacteria with 59 genera which showed reduced relative abundances and 21 genera that showed increased relative abundances due to fertilization. On the other hand, 30 genera from the phylum Actinobacteria increased their relative abundance and 3 decreased as a result of fertilization. In total 96 genera decreased their relative abundances and 66 increased due to fertilization. Specifically, 19 and 15 genera associated with N fixation decreased and increased their relative abundances, respectively (see Supplementary material for detailed list of N fixing genera).

**TABLE 4:** Bacterial and archaeal genera affected by fertilization.

	Number of genera affected by fertilization	
Phylum	Decreased relative abundance (number of N fixing genera)	Increased relative abundance (number of N fixing genera)
Acidobacteria	3 (0)	0 (0)
Actinobacteria	3 (0)	30 (6)
Bacteroidetes	7 (0)	2 (1)
Candidatus Tectomicrobia	0 (0)	1 (0)
Chloroflexi	0 (0)	2 (0)
Cyanobacteria	5 (2)	0 (0)
Deinococcus-Thermus	0 (0)	2 (0)
Elusimicrobia	1 (0)	0 (0)
Euryarchaeota	2 (2)	2 (2)
Fibrobacteres	1 (1)	0 (0)
Firmicutes	9 (3)	5 (1)
Nitrospinae	1 (0)	0 (0)
Nitrospirae	2 (1)	0 (0)
Planctomycetes	1 (0)	1 (0)
Proteobacteria	59 (10)	21 (5)
Spirochaetes	1 (0)	0 (0)
Thaumarchaeota	1 (0)	0 (0)
Total	96 (19)	66 (15)

When looking at the 30 top most abundant bacterial and archaeal genera (Table 5), genera that increased with fertilization included: *Bacillus*, *Arthrobacter*, *Rubrobacter*, *Conexibacter*, *Skermanella*, *Blastococcus*, *Microvirga*, *Streptomyces*, *Mesorhizobium*, *Hahella*, *Thermoleophilum*, *Lysobacter*, *Sphaerobacter*, *Devosia* and *Sinorhizobium*. In particular genera *Bacillus*, *Streptomyces* and *Devosia* showed a Fold change of relative abundance of fertilized vs. not fertilized > 2. On the other hand, genera that decreased include: *Rheinheimera*, *Massilia*, *Azoarcus*, *Duganella*, *Geobacter*, *Thiobacillus*, *Burkholderia*, *Janthinobacterium*, *Pirellula*, *Herbaspirillum*, *Xanthomonas*, *Zoogloea*, *Nitrospira*, *Limnobacter* and *Geothermobacter*. In particular, genera that showed a fold change lower than 0.5 were *Rheinheimera*, *Massilia*, *Duganella*, *Janthinobacterium, Herbaspirillum, Zoogloea* and *Limnobacter.*

**TABLE 5:** Relative abundance (%) of the 30 most abundant bacterial and archaeal genera significantly affected by the treatments.

Genera	No fertilization				Fertilization				Fold change Fertilized vs. Not fertilized
	No *Verticillium*		*Verticillium*		No *Verticillium*		*Verticillium*		
	No *Trichoderma*	*Trichoderma*	No *Trichoderma*	*Trichoderma*	No *Trichoderma*	*Trichoderma*	No Trichoderma	Trichoderma	
	F-V-T-	F-V-T+	F-V+T-	F-V+T+	F+V-T-	F+V-T+	F+V+T-	F+V+T+	
*Bacillus* ^1^	2.46 ± 0.06	3.40 ± 0.54	3.10 ± 0.53	2.90 ± 0.70	6.35 ± 0.71	9.52 ± 4.57	8.84 ± 1.12	5.30 ± 1.23	2.53
*Arthrobacter* ^1^	2.42 ± 0.13	1.94 ± 0.05	2.22 ± 0.62	1.63 ± 0.34	3.6 ± 0.625	3.75 ± 0.71	4.59 ± 0.59	4.04 ± 0.85	1.95
*Rheinheimera* ^1^	5.14 ± 2.30	2.70 ± 1.86	0.41 ± 0.30	5.02 ± 1.72	0.14 ± 0.03	0.56 ± 0.46	0.05 ± 0.00	0.10 ± 0.02	0.06
*Rubrobacter* ^1^	1.65 ± 0.11	1.28 ± 0.07	1.27 ± 0.14	1.22 ± 0.11	1.72 ± 0.18	1.59 ± 0.16	1.71 ± 0.16	1.47 ± 0.09	1.20
*Conexibacter* ^1^	1.50 ± 0.22	1.16 ± 0.13	0.90 ± 0.15	0.82 ± 0.24	1.88 ± 0.38	1.38 ± 0.22	2.20 ± 0.35	1.23 ± 0.12	1.53
*Massilia* ^1^	1.88 ± 0.29	0.919 ± 0.5	2.62 ± 0.73	4.39 ± 2.07	0.09 ± 0.03	0.14 ± 0.01	0.07 ± 0.02	0.31 ± 0.08	0.06
*Skermanella* ^1^	1.08 ± 0.14	1.22 ± 0.16	1.04 ± 0.10	1.08 ± 0.04	1.49 ± 0.17	1.22 ± 0.10	1.51 ± 0.06	1.51 ± 0.15	1.30
*Azoarcus* ^1^	1.81 ± 0.12	1.41 ± 0.19	1.19 ± 0.27	1.01 ± 0.17	0.60 ± 0.04	0.71 ± 0.14	0.77 ± 0.09	0.74 ± 0.07	0.52
*Blastococcus* ^1^	0.94 ± 0.09	0.84 ± 0.12	0.71 ± 0.21	0.74 ± 0.20	1.16 ± 0.20	0.92 ± 0.09	1.75 ± 0.24	1.14 ± 0.09	1.54
*Microvirga* ^1^	0.84 ± 0.02	0.82 ± 0.06	0.95 ± 0.14	0.7 ± 0.033	1.24 ± 0.17	0.991 ± 0.1	1.13 ± 0.17	1.13 ± 0.06	1.36
*Duganella* ^1^	1.67 ± 0.75	2.33 ± 2.19	1.00 ± 0.85	2.68 ± 1.10	0.02 ± 0.00	0.02 ± 0.00	0.02 ± 0.01	0.03 ± 0.01	0.01
*Streptomyces* ^1^	0.61 ± 0.05	0.44 ± 0.05	0.60 ± 0.02	0.39 ± 0.08	1.19 ± 0.15	1.34 ± 0.24	2.09 ± 0.59	1.03 ± 0.24	2.77
*Geobacter* ^1^	1.11 ± 0.10	1.61 ± 0.26	0.98 ± 0.17	0.81 ± 0.11	0.90 ± 0.20	0.72 ± 0.06	0.76 ± 0.01	0.66 ± 0.10	0.67
*Thiobacillus* ^1^	1.18 ± 0.06	1.03 ± 0.02	1.08 ± 0.06	0.81 ± 0.09	0.63 ± 0.06	0.79 ± 0.11	0.68 ± 0.05	0.69 ± 0.09	0.68
*Mesorhizobium* ^1^	0.62 ± 0.02	0.65 ± 0.06	0.67 ± 0.06	0.70 ± 0.06	0.85 ± 0.06	0.93 ± 0.05	0.83 ± 0.09	1.02 ± 0.12	1.37
*Burkholderia* ^1^	0.86 ± 0.03	0.77 ± 0.05	0.72 ± 0.01	0.64 ± 0.08	0.46 ± 0.05	0.42 ± 0.01	0.59 ± 0.11	0.58 ± 0.07	0.69
*Hahella* ^1^	0.54 ± 0.06	0.53 ± 0.02	0.50 ± 0.04	0.42 ± 0.05	0.73 ± 0.10	0.65 ± 0.02	0.73 ± 0.03	0.63 ± 0.07	1.38
*Janthinobacterium* ^1^	0.93 ± 0.25	0.28 ± 0.14	1.28 ± 0.63	1.89 ± 1.19	0.03 ± 0.01	0.04 ± 0.00	0.02 ± 0.00	0.09 ± 0.01	0.04
*Thermoleophilum* ^1^	0.55 ± 0.1	0.44 ± 0.03	0.34 ± 0.04	0.31 ± 0.06	0.71 ± 0.11	0.54 ± 0.09	0.87 ± 0.15	0.56 ± 0.06	1.63
*Lysobacter* ^1^	0.24 ± 0.04	0.22 ± 0.05	0.66 ± 0.04	0.31 ± 0.06	0.84 ± 0.11	0.67 ± 0.13	0.30 ± 0.09	0.93 ± 0.42	1.92
*Pirellula* ^1^	0.77 ± 0.06	0.59 ± 0.03	0.48 ± 0.10	0.53 ± 0.08	0.40 ± 0.04	0.43 ± 0.05	0.49 ± 0.02	0.47 ± 0.06	0.76
*Herbaspirillum* ^1^	0.31 ± 0.02	0.54 ± 0.16	1.36 ± 0.71	0.18 ± 0.03	0.20 ± 0.04	0.19 ± 0.04	0.16 ± 0.02	0.32 ± 0.10	0.36
*Xanthomonas* ^1^	0.59 ± 0.06	0.50 ± 0.15	0.438 ± 0.2	0.27 ± 0.04	0.18 ± 0.05	0.19 ± 0.03	0.25 ± 0.06	0.33 ± 0.09	0.53
*Sphaerobacter* ^1^	0.29 ± 0.03	0.28 ± 0.06	0.26 ± 0.01	0.22 ± 0.05	0.41 ± 0.07	0.47 ± 0.08	0.45 ± 0.06	0.35 ± 0.05	1.60
*Devosia* ^1^	0.19 ± 0.03	0.17 ± 0.02	0.19 ± 0.03	0.23 ± 0.04	0.39 ± 0.02	0.63 ± 0.17	0.28 ± 0.04	0.50 ± 0.14	2.31
*Sinorhizobium* ^1^	0.26 ± 0.04	0.23 ± 0.04	0.28 ± 0.06	0.24 ± 0.07	0.34 ± 0.03	0.29 ± 0.02	0.36 ± 0.05	0.56 ± 0.11	1.53
*Zoogloea* ^1^	0.06 ± 0.02	0.24 ± 0.07	2.10 ± 1.44	0.05 ± 0.01	0.01 ± 0.00	0.04 ± 0.00	0.01 ± 0.00	0.02 ± 0.01	0.03
*Nitrospira* ^1^	0.42 ± 0.02	0.33 ± 0.03	0.32 ± 0.04	0.36 ± 0.03	0.31 ± 0.04	0.29 ± 0.06	0.25 ± 0.00	0.23 ± 0.03	0.76
*Limnobacter* ^1^	0.83 ± 0.44	0.41 ± 0.16	0.17 ± 0.03	0.64 ± 0.24	0.04 ± 0.01	0.15 ± 0.08	0.02 ± 0.00	0.20 ± 0.16	0.20
*Geothermobacter* ^1^	0.50 ± 0.01	0.40 ± 0.01	0.33 ± 0.07	0.33 ± 0.07	0.18 ± 0.03	0.20 ± 0.03	0.28 ± 0.00	0.23 ± 0.04	0.57

^1^Factor Fertilization is significant for this genus. No other significant factors or interactions were found in a 3-way ANOVA including factors Fertilization, *Verticillium*, *Trichoderma* and all possible interactions. Values represent the mean ± standard error of the mean. See ANOVA table in Supplementary material.

**FIGURE 3:**
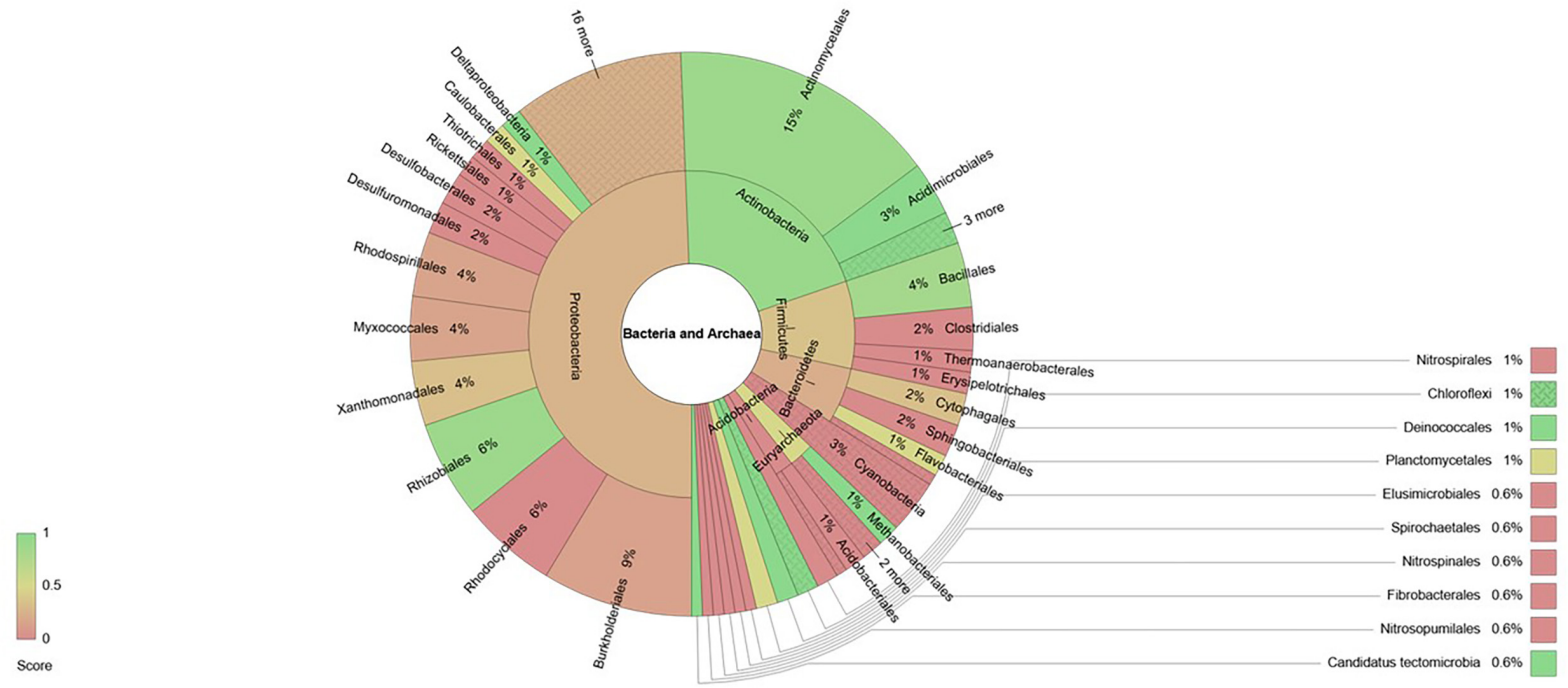
Krona diagram showing taxonomical distribution of bacteria and archaea genera affected by fertilization. Taxonomy levels shown are phylum and order. The color index is meant to indicate the proportion of genera within each taxonomical level whose relative abundance is decreased (red) or increased (green) by the fertilization treatment.

Furthermore, within each phylum the genera affected by fertilization belonged to orders that in some cases contained only positively affected genera, only negatively affected genera or a combination of both (Figure 3). In the case of the phylum Actinobacteria, the order Actinomycetales and Acidomicrobiales contained most of the genera that were found to have higher relative abundances due to fertilization. Within phylum Proteobacteria, the genera that were found to be more abundant due fertilization belonged mostly to the order Rhizobiales and Deltaproteobacteria, while orders Thiotrichales, Rickettsiales, Desulfobacterales, Desulfuromonadales and Rhodocyclales contained only genera that showed reduced relative abundances due to fertilization. On the other hand, in the phylum Firmicutes the order Bacillales contained mostly genera that were favored by fertilization, while Clostridiales, Thermoanaerobacterales and Erysipelotrichales contained only genera with reduced relative abundance due to fertilization. Bacteroidetes genera whose relative abundances were found to be reduced belonged mostly to order Sphingobacteriales. All 5 genera of Cyanobacteria found to be affected by fertilization showed reduced relative abundances and the same was true for the 3 genera of Acidobacteria. Within Euryarchaeota genera, the order Methanobacteriales contained two genera that were favored by fertilization while the genera that showed reduced relative abundances due to fertilization belonged to orders Methanosarcinales and Methanococcales.

The relative abundance of 9 fungal genera was affected by fertilization: *Rhizopus, Hygrocybe, Ramariopsis*, *Geoglossum, Chrysosporium*, and *Dactylellina* showed reduced relative abundances due to fertilization while *Acremonium, Ilyonectria* and *Inocybe* showed increased relative abundances (Table 6). T34 inoculation did not affect the relative abundance of any fungal genera. Moreover, genus *Verticillium* was found to be more relatively abundant when the inoculation with *V. dahliae* was performed.

**TABLE 6:** Relative abundance (%) of fungal genera significantly affected by the treatments.

Genera	No fertilization				Fertilization			
	No *Verticillium*		*Verticillium*		No *Verticillium*		*Verticillium*	
	No *Trichoderma*	*Trichoderma*	No *Trichoderma*	*Trichoderma*	No *Trichoderma*	*Trichoderma*	No *Trichoderma*	*Trichoderma*
	**F-V-T-**	**F-V-T+**	**F-V+T-**	**F-V+T+**	**F+V-T-**	**F+V-T+**	**F+V+T-**	**F+V+T+**
*Rhizopus* ^1^	8.36 ± 3.32	2.77 ± 1.44	11.12 ± 2.8	6.88 ± 1.81	0.91 ± 0.43	2.34 ± 0.83	0.79 ± 0.49	0.50 ± 0.37
*Hygrocybe* ^1^	3.55 ± 2.68	15.06 ± 3.43	3.71 ± 2.94	11.17 ± 5.66	0.11 ± 0.01	0.26 ± 0.14	0.2 ± 0.05	0.21 ± 0.03
*Ramariopsis* ^1^	0.59 ± 0.28	1.05 ± 0.45	0.31 ± 0.16	0.45 ± 0.16	0.02 ± 0	0.1 ± 0.06	0.08 ± 0.03	0.18 ± 0.07
*Acremonium* ^1^	0.71 ± 0.29	0.84 ± 0.13	0.70 ± 0.29	1.03 ± 0.18	2.09 ± 0.68	3.14 ± 0.85	2.96 ± 1.08	4.69 ± 2.54
*Geoglossum* ^1^	0.05 ± 0.03	0.06 ± 0.04	0 ± 0	0.03 ± 0.02	0 ± 0	0 ± 0	0.01 ± 0	0 ± 0
*Chrysosporium* ^1^	0.07 ± 0.03	0.07 ± 0.01	0.04 ± 0.01	0.04 ± 0.02	0.03 ± 0.01	0.01 ± 0.01	0.02 ± 0.01	0.01 ± 0
*Dactylellina* ^1^	0.17 ± 0.09	0.16 ± 0.08	0.1 ± 0.07	0.21 ± 0.16	0 ± 0	0.01 ± 0	0.01 ± 0	0.01 ± 0.01
*Ilyonectria* ^1^	0.24 ± 0.1	0.12 ± 0.04	0.07 ± 0.02	0.11 ± 0.04	0.81 ± 0.31	0.29 ± 0.21	1.42 ± 0.6	0.60 ± 0.31
*Inocybe* ^1^	0.22 ± 0.02	0.24 ± 0.07	0.27 ± 0.17	0.11 ± 0.02	0.77 ± 0.29	1.02 ± 0.55	0.91 ± 0.2	0.52 ± 0.31
*Verticillium* ^2^	0.09 ± 0.01	0.13 ± 0.02	1.04 ± 0.17	2.62 ± 0.09	0.11 ± 0.03	0.25 ± 0.18	4.94 ± 1.71	6.84 ± 2.88

^1^Factor Fertilization is significant for this genus. ^2^Factor *Verticillium* is significant for this genus. No other significant factors or interactions were found in a 3-way ANOVA including factors Fertilization, *Verticillium*, *Trichoderma* and all possible interactions. Values represent the mean ± standard error of the mean. See ANOVA table in Supplementary material.

According to FunGuild, those genera whose relative abundance increased in fertilized soils were classified as: *Rhizopus*, Pathotroph-Saprotroph; *Hygrocybe*, Saprotroph-Symbiotroph; *Ramariopsis*, Saprotroph; *Geoglossum*, Saprotroph; *Chrysosporium*, not present in FunGuild but according to Gopal et al. (2020) it is a Saprotroph and opportunistic human pathogen; and *Dactylellina*, Saprotroph. On the other hand, within fungal genera with reduced relative abundances due to fertilization *Acremonium* is Pathotroph-Saprotroph-Symbiotroph; *Ilyonectria*, Pathotroph; and *Inocybe*, Symbiotroph.

**FIGURE 4:**
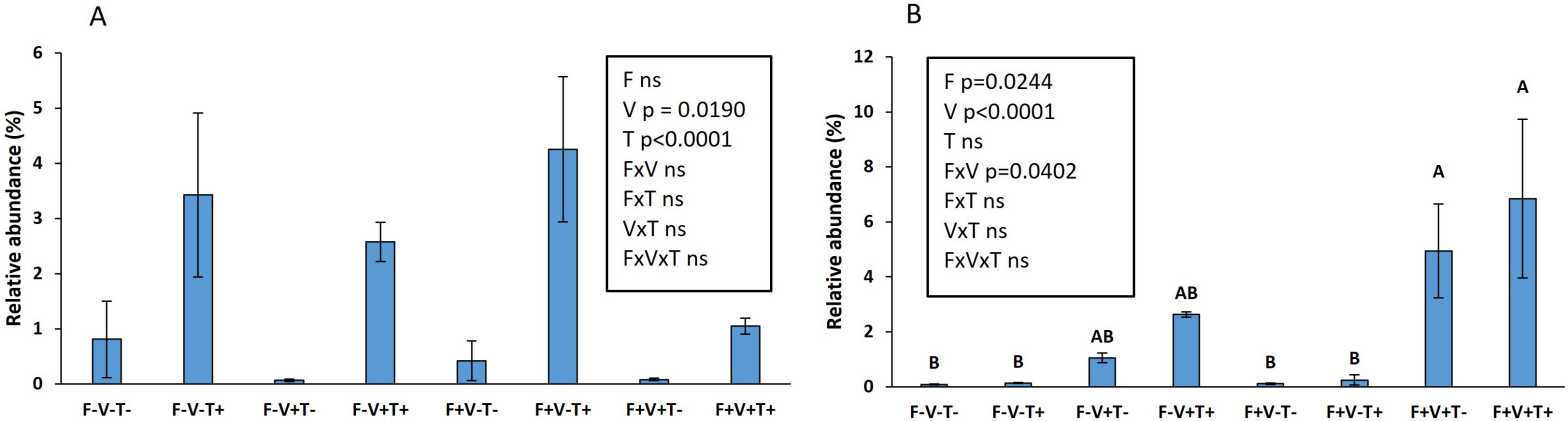
Effect of fertilization, *T. asperellum* T34 application and *V. dahliae* inoculation on the relative abundance of *T. asperellum* **(A)** and *V. dahliae* **(B)**. Fertilization: yes, F+; no, F-. *V. dahliae* inoculation: yes, V+; no, V-. *Trichoderma* application: yes, T+; no, T-. Means ± standard error of the means are shown. The number next to the name of the factor indicates the *p*-value.

When the effect of the 3 factors was studied specifically on the relative abundance of *T. asperellum* and *V. dahliae* at species level and without FDR correction, significant effects were observed (Figure 4). The relative abundance of *T. asperellum* was increased by the application of the biological control agent but was decreased by the inoculation with the pathogen while the factor fertilization was not significant (Figure 4A). Interestingly, *V. dahliae* relative abundance was dramatically increased due to the inoculation of the pathogen specifically when the soil was fertilized (Figure 4B).

Young leaves sampled 3 weeks after pathogen inoculation had higher concentrations of Ca, S and Mg if the plants had been treated with T34 (Table 7). In mature leaves sampled at the same time, fertilized plants had higher N content; plants treated with T34, had higher concentrations of P and lower Mg; plants inoculated with *V. dahliae* had lower levels of S. In the case of K, the plants treated with F+T-V- had lower concentrations than F-T+V+ and F-T+V- suggesting that T34 was more effective in increasing leaf K than mineral fertilization. Furthermore, plants treated with F-T+V+ had lower concentrations of Ca than F+T-V- and F-T-V+ (Table 8).

**TABLE 7:** Concentration of nutrients in young leaves sampled 3 weeks after pathogen inoculation.

Treatment	N (mg/g)	P (mg/g)	K (mg/g)	Ca (mg/g)	S (mg/g)	Mg (mg/g)	Fe (mg/g)
**No fertilization**							
V-T-	18.28 ± 2.09	1.74 ± 0.18	12.69 ± 1.03	3.72 ± 0.6	1.55 ± 0.16	0.72 ± 0.06	0.09 ± 0.02
V-T+	19.92 ± 1.78	2.10 ± 0.20	13.74 ± 1.18	5.73 ± 0.38	2.00 ± 0.22	1.07 ± 0.07	0.28 ± 0.62
V+T-	20.18 ± 4.03	1.84 ± 0.26	13.30 ± 0.84	3.64 ± 1.24	1.58 ± 0.25	0.70 ± 0.23	0.12 ± 0.05
V+T+	21.84 ± 2.96	2.29 ± 0.19	11.84 ± 1.08	5.96 ± 1.38	1.69 ± 0.15	1.20 ± 0.19	0.14 ± 0.16
**Fertilization**							
V-T-	26.10 ± 3.00	2.33 ± 0.25	13.24 ± 0.75	2.44 ± 0.88	1.87 ± 0.25	0.73 ± 0.13	0.82 ± 0.06
V-T+	24.00 ± 3.42	2.10 ± 0.26	13.23 ± 1.03	4.49 ± 0.93	2.10 ± 0.20	1.04 ± 0.14	0.29 ± 0.04
V+T-	17.56 ± 2.27	1.78 ± 0.31	12.67 ± 0.64	2.94 ± 0.94	1.67 ± 0.23	0.72 ± 0.21	0.05 ± 0.03
V+T+	20.78 ± 2.31	2.19 ± 0.13	12.29 ± 0.42	4.33 ± 0.45	2.18 ± 0.12	0.87 ± 0.05	0.19 ± 0.03
**ANOVA**							
Fertilization	n.s	n.s.	n.s.	n.s.	n.s.	n.s.	n.s.
*Trichoderma*	n.s.	n.s.	n.s.	0.006	0.017	0.002	n.s.
Fertilizacion × *Trichoderma*	n.s.	n.s.	n.s.	n.s.	n.s.	n.s.	n.s.
*Verticillium*	n.s.	n.s.	n.s.	n.s.	n.s.	n.s.	n.s.
Fertilization × *Verticillium*	n.s.	n.s.	n.s.	n.s.	n.s.	n.s.	n.s.
*Trichoderma* × *Verticillium*	n.s.	n.s.	n.s.	n.s.	n.s.	n.s.	n.s.
Fertilization × *Trichoderma* × *Verticillium*	n.s.	n.s.	n.s.	n.s.	n.s.	n.s.	n.s.

Values represent the mean ± standard error of the mean.

**TABLE 8:** Concentration of nutrients in mature leaves sampled 3 weeks after pathogen inoculation.

Treatment	N (mg/g)	P (mg/g)	K (mg/g)	Ca (mg/g)	S (mg/g)	Mg (mg/g)	Fe (mg/g)
**No fertilization**							
V-T-	23.20 ± 1.17	1.90 ± 0.15	12.10 ± 0.39abc	8.20 ± 0.71ab	2.34 ± 0.40	1.65 ± 0.07	0.08 ± 0.03
V-T+	25.54 ± 1.76	2.31 ± 0.14	15.83 ± 1.01a	4.08 ± 1.22ab	2.52 ± 0.16	1.10 ± 0.20	0.06 ± 0.09
V+T-	23.72 ± 0.96	2.26 ± 0.18	11.02 ± 0.56bc	8.46 ± 0.56a	1.04 ± 0.59	1.57 ± 0.06	0.19 ± 0.06
V+T+	20.88 ± 1.57	2.48 ± 0.31	14.97 ± 1.53ab	3.55 ± 0.93b	0.42 ± 0.59	1.22 ± 0.32	0.07 ± 0.03
**Fertilization**							
V-T-	32.12 ± 3.2	1.93 ± 0.38	10.21 ± 0.94c	8.56 ± 0.80a	3.36 ± 0.72	1.90 ± 0.12	0.09 ± 0.02
V-T+	31.88 ± 3.31	2.30 ± 0.22	12.83 ± 1.00abc	7.97 ± 1.04ab	2.64 ± 0.53	1.62 ± 0.21	0.12 ± 0.04
V+T-	29.92 ± 2.82	1.88 ± 0.19	13.09 ± 0.72abc	7.70 ± 1.03ab	1.08 ± 0.56	1.49 ± 0.13	0.12 ± 0.04
V+T+	32.72 ± 1.89	2.56 ± 0.21	13.90 ± 1.29abc	5.85 ± 1.13ab	1.46 ± 0.39	1.22 ± 0.21	0.19 ± 0.08
**ANOVA**							
Fertilization	0.000	n.s.	n.s.	0.046	n.s.	n.s.	n.s.
*Trichoderma*	n.s.	0.014	0.000	0.000	n.s.	0.012	n.s.
Fertilizacion × *Trichoderma*	n.s.	n.s.	n.s.	0.024	n.s.	n.s.	n.s.
*Verticillium*	n.s.	n.s.	n.s.	n.s.	0.000	n.s.	n.s.
Fertilization × *Verticillium*	n.s.	n.s.	0.040	n.s.	n.s.	n.s.	n.s.
*Trichoderma* × *Verticillium*	n.s.	n.s.	n.s.	n.s.	n.s.	n.s.	n.s.
Fertilization × *Trichoderma* × *Verticillium*	n.s.	n.s.	n.s.	n.s.	n.s.	n.s.	n.s.

Values represent the mean ± standard error of the mean. Numbers followed by different letters are significantly different (*P* < 0.05, Tukey’s test).

Young leaves sampled 3 months after pathogen inoculation had higher concentrations of N, P and S but lower levels of Ca if the plants had been fertilized (Table 9).

**TABLE 9:** Concentration of nutrients in young leaves sampled 3 months after pathogen inoculation.

Treatment	N (mg/g)	P (mg/g)	K (mg/g)	Ca (mg/g)	S (mg/g)	Mg (mg/g)	Fe (mg/g)
**No fertilization**							
V-T-	11.16 ± 1.83	1.45 ± 0.21	15.44 ± 0.60	6.11 ± 0.71	1.26 ± 0.12	0.70 ± 0.02	0.02 ± 0.01
V-T+	12.34 ± 2.55	1.49 ± 0.20	16.06 ± 0.50	6.49 ± 1.03	1.39 ± 0.14	0.77 ± 0.04	0.03 ± 0.00
V+T-	11.48 ± 2.79	1.34 ± 0.22	16.04 ± 0.77	5.97 ± 0.78	1.19 ± 0.18	0.73 ± 0.03	0.03 ± 0.00
V+T+	11.94 ± 2.38	1.25 ± 0.20	14.60 ± 0.71	7.32 ± 1.14	1.24 ± 0.11	0.73 ± 0.03	0.03 ± 0.01
**Fertilization**							
V-T-	18.82 ± 1.65	2.14 ± 0.17	17.89 ± 0.68	3.62 ± 0.54	1.76 ± 0.11	0.74 ± 0.05	0.04 ± 0.00
V-T+	18.30 ± 2.01	1.97 ± 0.18	16.34 ± 1.02	4.45 ± 0.81	1.61 ± 0.13	0.68 ± 0.03	0.03 ± 0.01
V+T-	17.92 ± 1.48	1.79 ± 0.12	15.63 ± 0.99	5.14 ± 1.13	1.47 ± 0.12	0.76 ± 0.03	0.04 ± 0.00
V+T+	18.53 ± 2.43	1.84 ± 0.18	15.47 ± 0.34	5.13 ± 0.38	1.56 ± 0.19	0.74 ± 0.05	0.04 ± 0.01
**ANOVA**							
Fertilization	0.000	0.000	n.s.	0.001	0.001	n.s.	n.s.
*Trichoderma*	n.s.	n.s.	n.s.	n.s.	n.s.	n.s.	n.s.
Fertilizacion × *Trichoderma*	n.s.	n.s.	n.s.	n.s.	n.s.	n.s.	n.s.
*Verticillium*	n.s.	n.s.	n.s.	n.s.	n.s.	n.s.	n.s.
Fertilization × *Verticillium*	n.s.	n.s.	n.s.	n.s.	n.s.	n.s.	n.s.
*Trichoderma* × *Verticillium*	n.s.	n.s.	n.s.	n.s.	n.s.	n.s.	n.s.
Fertilization × *Trichoderma* × *Verticillium*	n.s.	n.s.	n.s.	n.s.	n.s.	n.s.	n.s.

Values represent the mean ± standard error of the mean.

Mature leaves sampled 3 months after pathogen inoculation had higher concentrations of N, K and S if the plants had been fertilized (Table 10). Inoculation with the pathogen led to higher concentration of Fe in those leaves.

**TABLE 10:** Concentration of nutrients in mature leaves sampled 3 months after pathogen inoculation.

Treatment	N (mg/g)	P (mg/g)	K (mg/g)	Ca (mg/g)	S (mg/g)	Mg (mg/g)	Fe (mg/g)
**No fertilization**							
V-T-	12.48 ± 1.52	1.29 ± 0.11	10.14 ± 0.91	8.41 ± 1.58	1.35 ± 0.28	0.84 ± 0.21	0.05 ± 0.02
V-T+	11.74 ± 0.86	1.73 ± 0.22	11.16 ± 1.07	9.35 ± 0.98	1.29 ± 0.10	0.74 ± 0.09	0.03 ± 0.02
V+T-	12.38 ± 1.38	1.41 ± 0.16	10.45 ± 0.85	8.74 ± 1.65	1.26 ± 0.24	0.87 ± 0.19	0.10 ± 0.04
V+T+	11.10 ± 0.96	1.64 ± 0.24	9.93 ± 1.00	9.15 ± 1.42	1.17 ± 0.11	0.73 ± 0.12	0.08 ± 0.03
**Fertilization**							
V-T-	17.20 ± 1.49	1.45 ± 0.2	12.21 ± 0.82	7.11 ± 1.27	1.44 ± 0.12	0.61 ± 0.10	0.04 ± 0.04
V-T+	14.52 ± 0.93	1.73 ± 0.21	11.42 ± 0.99	8.23 ± 1.01	1.36 ± 0.11	0.66 ± 0.12	0.03 ± 0.00
V+T-	17.00 ± 1.03	1.99 ± 0.12	13.41 ± 1.02	9.68 ± 0.99	1.51 ± 0.13	0.84 ± 0.12	0.04 ± 0.01
V+T+	17.72 ± 1.04	1.89 ± 0.20	11.86 ± 0.83	9.87 ± 1.32	1.61 ± 0.13	0.98 ± 0.10	0.08 ± 0.03
**ANOVA**							
Fertilization	0.000	n.s.	0.010	n.s.	0.047	n.s.	n.s.
*Trichoderma*	n.s.	n.s.	n.s.	n.s.	n.s.	n.s.	n.s.
Fertilizacion × *Trichoderma*	n.s.	n.s.	n.s.	n.s.	n.s.	n.s.	n.s.
*Verticillium*	n.s.	n.s.	n.s.	n.s.	n.s.	n.s.	0.049
Fertilization × *Verticillium*	n.s.	n.s.	n.s.	n.s.	n.s.	n.s.	n.s.
*Trichoderma* × *Verticillium*	n.s.	n.s.	n.s.	n.s.	n.s.	n.s.	n.s.
Fertilization × *Trichoderma* × *Verticillium*	n.s.	n.s.	n.s.	n.s.	n.s.	n.s.	n.s.

Values represent the mean ± standard error of the mean.

## 4 Discussion

### 4.1 Lack of disease symptoms

It is known that the disease produced by *V. dahlia*e has two distinct phases which consist of an initial biotrophic phase, in which symptoms are not yet evident, and a necrotrophic phase, that involves wilt symptoms when colonization is widespread and which occurs as a consequence of impaired vascular transportation (Scholz et al., 2018). No wilt or defoliation symptoms of disease produced by *V. dahliae* inoculation were found in this experiment, even though the olive tree variety used is highly susceptible and the virulent defoliating pathotype of the pathogen was used (Garcia-Ruiz et al., 2014). Disease symptoms did not develop even after several reinoculations with the pathogen but the relative abundance of *Verticillium dahliae* sequencing reads in soil samples increased due to the application of the pathogen, particularly in fertilized pots.

One explanation for the lack of symptoms development could be the high temperatures recorded during the assay, in particular during the summer, since it has been shown that temperatures above 25 °C are usually detrimental to the development of the disease by *V. dahliae* and could slow or stop the biotrophic host-pathogen phase and difficult its transition to the necrotrophic phase (Calderón et al., 2014). Moreover, variations in climatic conditions, such as temperature, along the year can slow the disease progress in comparison with constant temperatures favorable to the fungus (Calderón et al., 2014). Furthermore, the soil used in the pots could have had unexpected suppressive effects (Tienda et al., 2025). In this sense a reduction of *Verticillum dahliae* disease severity has been observed as a result of using substrates based on compost to grow plants (Avilés and Borrero, 2017). Interestingly, the significant reduction on S content on young leaves inoculated with Vd could be attributed to competition of host and pathogen in the early stage of the interaction (Romanyà et al., 2019).

Between 1.2 a 54 microesclerotia per gram of soil were found in samples collected from stablished olive orchards affected by Verticillium wilt (López-Escudero and Blanco-López, 2005). Moreover, a natural infestation of soil with *V. dahliae* was determined to be 5.5 microsclerotia per gram while 35 microsclerotia per gram of soil was considered to be a high inoculum density (Mulero-Aparicio et al., 2020). In this sense, the levels of microsclerotia found in the present work could be considered in the range of a naturally occurring infection.

### 4.2 *Trichoderma* effects on microsclerotia

Even though the conditions in this assay were not favorable to study the biological control activity of *T. asperellum* strain T34 on *V. dahliae*, due to the lack of plant symptoms observed, it was possible to prove the effect of the biological control agent in reducing the concentration of microsclerotia in the potted soil. This reduction was quite notable (70–80%) and was independent of the presence of mineral fertilization. Other authors found that soil inoculated with the fungus *Talaromyces flavus* reduced the germinability of *V. dahliae* microsclerotia which had been buried in treated soil for 14 days and electronic microscopy revealed that microsclerotia were colonized by *T. flavus* (Fahima et al., 1992). We hypothesize that mycoparasitism of *V. dahliae* microsclerotia by T34 hyphae could play an important role in the biological control of Verticillium wilt disease. In this sense, a grape marc compost that showed a high capacity to inhibit microsclerotia viability, also greatly reduced the severity produced by *V. dahliae* in olive plants (Varo-Suárez et al., 2018). A survey that studied fungi colonizing microsclerotia in a wide variety of habitats, showed that *Trichoderma koningii*, *Fusarium oxysporum* and *Alternaria alternata* were the most frequently isolated from microsclerotia buried in the soil and it was hypothesized that these species possess a greater affinity for microsclerotia of *V. dahliae* than other species present in the soil (Grunden et al., 2001). In the past, there has been research efforts to use mycoparasites for the control of *V. dahliae* by using isolates of the genus *Talaromyces*, *Trichoderma* and *Gliocladium* (Grunden et al., 2001). Interestingly, *T. asperellum* strain T25 has shown promising results in terms of controlling the disease produced by *V. dahliae* in olive trees since its application delayed the time of first symptom detection and also the disease severity (Carrero-Carrón et al., 2016).

### 4.3 Effects of *Trichoderma* on *V. dahliae* and viceversa

Even though T34 application reduced the number of microsclerotia per gram of soil, its application did not have a significant effect on the relative abundance of *Verticillium dahliae* sequencing reads in soil samples. T34 has been reported to control the disease caused by *Fusarium oxysporum* f.sp. *lycopersici* on tomato plants in pot experiments and in that case T34 was shown to reduce the population of *F. oxysporum.* Moreover, the population of T34 increased in the presence of the pathogen, probably as a consequence of hyperparasitism (Segarra et al., 2010).

On the other hand, when *V. dahliae* was inoculated, the relative abundance of T34 decreased significantly. Interestingly, strains Bt2, Bt3 and T25 of *T. asperellum* which were able to overgrow the colonies of several *V. dahliae* isolates *in vitro*, showed diverse degree of susceptibility to *V. dahliae*-secreted compounds since growth of Bt2 was reduced whereas that of Bt3 and T25 was not affected (Carrero-Carrón et al., 2016). It is noteworthy that *V. dahliae* has been shown to inhibit fungal growth *in vitro*, to produce antibiotics, detoxify antifungal compounds, and act as a mycoparasite (Barron and Fletcher, 1970; Grunden et al., 2001).

### 4.4 Effect of fertilization on microbial communities

Despite the limitations of a single point sampling for microbiome studies, which has previously been reported, many publications about soil microbiome still rely on sampling at a single point in time (Geisen and Stefan, 2021). This is a limitation of this experiment, since it is clear that seasonal factors are important in shaping soil biodiversity (Boutin and Laforest-Lapointe, 2025). In this study, the application of mineral fertilization, treatment with *T. asperellum* strain T34 and inoculation with the pathogen *V. dahliae* did not have an impact on bacterial and fungal diversity indices. The factor fertilization was the only that affected significantly the fungal and bacterial communities at OTU level when studied by PCoA and Permanova, while the effect of *Trichoderma* and *Verticillium* inoculation was not significant. It is known that plants have a profound effect on the microbiome of the rizhosphere but soil and management practices also have an important role (Bulgarelli et al., 2013; Mishra et al., 2022). Interestingly, similarly to what happens in our study, Eo and Park (2016) did not find a significant effect of mineral fertilization on diversity indices of soil cultivated with pepper plants, while the effect of fertilization varied at genus level. On the other hand, in a review of studies that experimentally exposed microbial communities to various disturbances, more than 80% of the mineral fertilization studies found significant effects of disturbance on microbial composition (Allison and Martiny, 2008). In addition (at 120 kg N ha^–1^, similar to the 140 kg used in our study) resulted in a significant shift in bacterial community composition and a decrease in bacterial OTU richness in surface soil in in a grassland fertilization experimental field (Zeng et al., 2016). Long-term use of inorganic nitrogen (N) fertilization influenced the bacterial community in a black soil in China: N addition consistently decreased bacterial diversity and altered bacterial community composition, by increasing the relative abundance of Proteobacteria, and decreasing that of Acidobacteria and Nitrospirae (Zhou et al., 2017).

It is noteworthy that in a meta-analysis on changes in bacterial community under long-term mineral fertilization, bacterial taxonomic diversity was decreased by N fertilization alone but was increased by NPK fertilization in a variety of soil chemical and physical properties (Dai et al., 2018). Nitrogen fertilization increased the relative abundance of Proteobacteria and Actinobacteria, but reduced the abundance of Acidobacteria (Dai et al., 2018). However, while in our study an increase in the relative abundance of Actinobacteria associated with mineral fertilization was observed, the relative abundance of Proteobacteria slightly decreased. In addition, a study on 25 globally distributed grassland sites showed consistent alterations in microbial communities as a consequence of N and P addition (Leff et al., 2015). In particular, nutrient application decreased the relative abundance of mycorrhizal fungi, methanogenic archaea, and oligotrophic bacterial taxa, while increased the relative abundances of fast growth copiotrophic bacterial taxa (Leff et al., 2015). In our study, we did not find a significant effect of fertilization on Glomeromycota. While the relative abundance of Actinobacteria (considered copiotrophic) increased as a result of fertilization, we did not observe a significant effect on the oligotrophic Acidobacteria phylum. On the contrary, in our study, relative abundance of Proteobacteria, which are usually considered to be copiotrophic, slightly decreased with fertilizer application.

There have been attempts to classify high level bacterial taxa into ecologically meaningful categories such as copiotrophic and oligotrophic in relation to C and N availability. A negative correlation of the abundance of Acidobacteria and a positive relationship for both Bacteroidetes and Betaproteobacteria and C amendment level were found (Fierer et al., 2007). On the other hand, Gammaproteobacteria and Actinobacteria increased with N inputs while Acidobacteria, Cyanobacteria, and Nitrospira decreased with N input rates (Ramirez et al., 2010). In our case, the metabollicaly active (copiotrophic) pyllum Bacteroidetes was not affected by fertilization.

It is remarkable that the most abundant bacterial phylum in the rhizosphere (Proteobacteria, Actinobacteria and Firmicutes) were significantly affected by fertilization in our study. Proteobacteria and Actinobacteria are the most abundant phyla in microbiome studies on olive orchard soils (Fausto et al., 2018; Pathan et al., 2021). In fact, 59 genera of Proteobacteria (including 10 N fixing genera) had their relative abundances decreased due to fertilization while 21 (including 5 N fixing bacteria) increased due to fertilization. Moreover, the relative abundance of 30 Actinobacterial genera increased, including 6 N fixing genera, while only 3 Actinobacterial genera decreased due to fertilization. Overall, there was not a clear effect of mineral fertilization on the relative abundances of N fixing bacteria since the relative abundances of 19 and 15 genera associated with N fixation were decreased and increased, respectively, due to fertilization.

Firmicutes thrive in carbon-rich soils (Cesarano et al., 2017). In 28 North-American soils, inorganic N increased Actinobacteria and Firmicutes, but reduced Acidobacteria and Verrucomicrobia (Ramirez et al., 2012). Nitrospirae bacteria, dominant nitrite oxidizers in various environments including olive orchards are considered oligotrophic taxa and usually decline with nitrogen addition, as happened in the present study (Caliz et al., 2015; Eo and Park, 2016; Fujitani et al., 2020; Beltran-Garcia et al., 2021). Nitrospinae, which are marine nitrite-oxidizing bacteria decreased with high N organic fertilization and in our study were completely suppressed by fertilization (Spieck et al., 2014; Wang M. et al., 2022). Tectomicrobia, known for bioactive compound synthesis in sponges, increased under mineral fertilization despite previously described negative correlations with N and P soil content (Wilson et al., 2014; Saravanakumar et al., 2016; Wang Q. et al., 2022). Fibrobacteres, cellulose degraders, decreased with fertilization (Ransom-Jones et al., 2012; Jewell et al., 2013). Tenericutes, with limited rhizospheric roles but involved in polyol synthesis, also declined (Sun et al., 2022). Elusimicrobia, found in insect guts and soils, were reduced by fertilization in our study and have been previously shown to decrease by N fertilization while showing a positive correlation with available soil K (Zhang et al., 2019; Méheust et al., 2020; Yu et al., 2023). Spirochaetes, spiral-shaped bacteria with diverse lifestyles, increased in corn soils treated with manure and fertilizer, contrary to our findings (Wolters et al., 2018; Hallmaier-Wacker et al., 2019).

Considering the 30 top most abundant bacteria and archaea genera affected by fertilization, there was an increase in the abundance of genera of aerobic chemoheterotrophs and decomposers, such as *Arthrobacter, Bacillus*, *Blastococcus, Conexibacter, Lysobacter, Skermanella, Streptomyces;* and the thermophilic *Rubrobacter, Sphaerobacter* and *Thermoleophilum;* with the exception of the genus *Massilia* which decreased. These genera harbor broad enzyme repertoires that contribute to organic-matter turnover and degradation of complex metabolites and competitive traits such as sporulation and secondary metabolites synthesis (Yakimov et al., 2003; Pati et al., 2010; Zhu et al., 2015; Expósito et al., 2015; Chater, 2016; Castro et al., 2019; Saxena et al., 2020; Gushgari-Doyle et al., 2022; Lei et al., 2023; Amirhosseini et al., 2025; Sbissi et al., 2025). On the other hand, fertilization increased the abundance of genera related to classical rhizobial symbionts such as *Mesorhizobium* and *Sinorhizobium* and other nodulating bacteria with nitrogen-fixation potential such as some *Microvirga* and *Devosia* species, whereas associative/freeliving N fixers such as *Azoarcus* and *Herbaspirillum* decreased, as did the functionally diverse *Burkholderia* which includes some N-fixing species (Coenye and Vandamme, 2003; Laranjo et al., 2014; Toro et al., 2017; Jiménez-Gómez et al., 2019; Raittz et al., 2021; Pedrolo et al., 2023; Tian et al., 2024). On the other hand, there was a reduction in the abundance of chemolithotrophic and redox-specialist guilds. The fertilized soils showed consistent decreases in sulfur oxidizers (*Thiobacillus* and *Limnobacter*), as well as iron reducers (*Geobacter, Geothermobacter*) (Chen et al., 2016; Hillary et al., 2018; Reguera and Kashefi, 2019; Lopez-Fernandez et al., 2023). Concomitantly, there was a decline of nitrifiers (*Nitrospira*, canonical nitrite oxidizers) (Meng et al., 2023). Several taxa that declined such as *Rheinheimera*, *Duganella*, *Zoogloea*, *Janthinobacterium* and *Pirellula* are frequently linked to aquatic environments which are potentially oligotrophic, with the exception of *Hahella* that increased with fertilization (Glöckner et al., 2003; Chen et al., 2010; Haack et al., 2016; Muller et al., 2017; He et al., 2022; Zhao et al., 2022). Notably, the decrease in the plant-pathogenic genus *Xanthomonas* coincided with the rise of antagonistic taxa such as *Bacillus*, *Lysobacter*, and *Streptomyces* (Huang et al., 2015).

Fertilization negatively affected the relative abundance of Mucoromycotina. Mucoromycotina “fine root endophytes,” are soil fungi that form endosymbioses with a wide range of plants and are distinct from the arbuscular mycorrhizal fungi (which belong to the Glomeromycota) in that they transfer a significant amount of nitrogen to their host plant (Sinanaj et al., 2021). Interestingly, in our study, the relative abundance of Glomeromycota was not affected by fertilization.

In fertilized soils, the inoculation with *V. dahliae* and/or *Trichoderma* had a negative effect on Blastocladiomycota. The zoosporic fungi of Blastocladiomycota are important components in freshwater ecosystems, decomposing of organic matter and/or parasiting several hosts (Jerônimo and Pires-Zottarell, 2019).

Furthermore, in our study fertilization had an effect on the relative abundances of some fungal taxa at genus level. There was not a clear tendency on whether certain groups were favored or inhibited by fertilization according to their phylum and trophic mode (FunGuild). It is remarkable, however that relative abundance of the nematode trapping genus *Dactylellina* decreased and the plant pathogenic genus *Ilyonectria* increased due to fertilization (Jiang et al., 2016).

### 4.5 Effect of *Trichoderma asperellum* strain T34 and *Verticillium* on microbial communities

Manufacturers of plant protection products based on microorganism must prove the safety of the product in terms of toxicological and ecotoxicological profiles. There have been some reviews raising concerns about the potential negative effects of biological control agents such as *Trichoderma* spp. on non-target organisms. For instance, Brimner and Boland (2003) mentioned in their review that non-target effects could include mycoparasitism of mycorrhizae and reductions in plant root colonization by mycorrhizal fungi and nodulation by *Rhizobium* spp.

Repeated application of *T. asperellum* strain T34 did not affect significantly the diversity of fungi and bacteria nor the relative abundances of phyla and genera. In this sense, the application of the antagonistic strain of *Trichoderma atroviride* strain I-1237 slightly modified the microbial diversity only for a short period of time, studied by means of terminal restriction fragment length polymorphism method of 18S and 16S rRNA genes and 9 months after the inoculation, differences between control and inoculated soils were no longer found (Cordier and Alabouvette, 2009).

Given that some detrimental unintentional effects produced by *Trichoderma* spp. isolates appear in the literature, it is important that studies on non-target effects of microbial PPP are performed for each strain and no conclusions on safety are taken at genus or species level (Jangir et al., 2019).

Since there is a trend on the increase of use of microbial PPP and very likely there are going to be interactions with other beneficial microorganisms applied to improve crop fertilization such as N-fixing microorganisms (*Azospirillum* spp., *Rhizobium* spp.) and those that increase P availability (such as mycorrhizae), the compatibility of PPP with microbial biostimulants is an important trait to be considered. In, this sense, the inoculation with the biostimulant *Azospirillum brasilense* Sp245 and biocontrol strains *Pseudomonas fluorescens* WCS 365 and *Trichoderma harzianum* T12 did not show negative effects on arbuscular mycorrhizal fungi establishment in the rhizosphere of maize plants (Vázquez et al., 2000).

Similarly to what happens in our study, the introduction of the pathogen *V. dahliae* in two olive cultivars did not result in significant alterations in the structure and functionality of soil microbial communities (Fernández-González et al., 2020).

### 4.6 Effect of fertilization on *V. dahliae*

Fertilization has an impact on the progression of vascular diseases which has been reported in various crops. In particular, high N fertilization in irrigated olive tree orchards is expected to stimulate the incidence and severity of *V. dahliae* wilt (López-Escudero and Mercado-Blanco, 2011). In this sense, in our experiment both the number of microsclerotia per gram of soil and the relative abundance of *V. dahliae* increased significantly due to mineral fertilization which included both nitrate and amonia.

The application of T34 increased the concentration of Ca, S and Mg in young leaves and of P in mature leaves. This effect was not observed 3 months after the application suggesting that the beneficial effects last for a certain amount of time after the application. Interestingly, Mg concentration in mature leaves was lower in the presence of T34 suggesting that there might be a stimulation of the mobilization of this element from mature to young leaves in the presence of T34. T34 application to the root system has been shown to increase the concentrations of nutrients such as Ca, Mg, Mn, B, and Si in tomato leaves grown in pots (Bidellaoui et al., 2019).

Interestingly, inoculation with the pathogen *V. dahliae* resulted in lower concentrations of S in the leaves 3 weeks after pathogen inoculation. It has been shown that S plays an important role in tomato disease resistance against *V. dahliae* by synthesis of sulfur containing defense compounds (Fu et al., 2016). Moreover, there seems to be an important plant-pathogen competition for available S in the initial phases of the interaction (Romanyà et al., 2019).

Inoculation with the pathogen led to higher concentration of Fe in mature leaves sampled 3 months after pathogen inoculation. Interestingly, addition of FeEDDHA to eggplants infected with *V*. *dahliae* significantly reduced disease severity in calcareous soil, suggesting an implication or Fe homeostasis in pathogenicity of this pathogen (Barash et al., 1988).

## 5 Conclusion

The treatment of the pots with the biological control agent *T. asperellum* strain T34 effectively reduced the amount of *V. dahliae* microsclerotia suggesting a promising alternative to chemical fumigation. Moreover, it did not affect the diversity of bacteria and fungi in the rhizospheric soil of olive trees. On the other hand, mineral fertilization doubled the amount of microsclerotia in soil and drastically increased the relative abundance of *V. dahliae* reads. Furthermore, fertilization had a significant effect on microbial communities, affecting the relative abundance of 162 and 9 genera of bacteria and fungi, respectively. Interestingly, fertilization did not have an effect on the phylum Glomeromycota and bacterial genera affected by fertilization were not specifically associated to N fixing or non-N fixing bacteria.

Taken together, those results suggest that mineral fertilization has a much more profound impact on the relative abundance of microorganisms than the introduction of biological control agents such as *T. asperellum* strain T34.

## Data Availability

The datasets presented in this study can be found in online repositories. The names of the repository/repositories and accession number(s) can be found at: https://www.ncbi.nlm.nih.gov/, PRJNA628525.

## Footnotes

1
https://www.glbrc.org/data-and-tools/glbrc-data-sets/ribosomal-database-project

2
www.ncbi.nlm.nih.gov
